# Supplementary material for: An open-source framework for large-scale, flexible evaluation of biomedical text mining systems
Source: J Biomed Discov Collab. 2008 Jan 29;3:1. doi: 10.1186/1747-5333-3-1 (PMC2276192; doi:10.1186/1747-5333-3-1)
Supplement: Additional file 1 — Evaluation platform source code. The additional file contains the software evaluation framework discussed in this paper. The most current release can be downloaded from SourceForge.net [1]. See the accompanying README file for instructions on installation and use. Also included in the distribution is a collection of UIMA wrappers for some commonly used BioNLP tools and annotated corpora. [file 1747-5333-3-1-S1.zip › bionlp_uima_repository-1.0.1/docs/api/src-html/edu/uchsc/ccp/uima/annotation/syntactic/CCPTokenAnnotation.html]

```
001    
002    
003    /* First created by JCasGen Tue Aug 28 16:44:10 MDT 2007 */
004    package edu.uchsc.ccp.uima.annotation.syntactic;
005    
006    import org.apache.uima.jcas.JCas;
007    import org.apache.uima.jcas.JCasRegistry;
008    import org.apache.uima.jcas.cas.FSArray;
009    import org.apache.uima.jcas.cas.TOP_Type;
010    
011    import edu.uchsc.ccp.uima.annotation.CCPSyntacticAnnotation;
012    
013    
014    /** This annotation type is used to mark up tokens.
015     * Updated by JCasGen Tue Aug 28 16:44:10 MDT 2007
016     * XML source: /home/williamb/eclipse/RELEASED_SOURCE_CODE/descriptors/typeSystem/CCPTypeSystem.xml
017     * @generated */
018    public class CCPTokenAnnotation extends CCPSyntacticAnnotation {
019      /** @generated
020       * @ordered 
021       */
022      public final static int typeIndexID = JCasRegistry.register(CCPTokenAnnotation.class);
023      /** @generated
024       * @ordered 
025       */
026      public final static int type = typeIndexID;
027      /** @generated  */
028      public              int getTypeIndexID() {return typeIndexID;}
029     
030      /** Never called.  Disable default constructor
031       * @generated */
032      protected CCPTokenAnnotation() {}
033        
034      /** Internal - constructor used by generator 
035       * @generated */
036      public CCPTokenAnnotation(int addr, TOP_Type type) {
037        super(addr, type);
038        readObject();
039      }
040      
041      /** @generated */
042      public CCPTokenAnnotation(JCas jcas) {
043        super(jcas);
044        readObject();   
045      } 
046    
047      /** @generated */  
048      public CCPTokenAnnotation(JCas jcas, int begin, int end) {
049        super(jcas);
050        setBegin(begin);
051        setEnd(end);
052        readObject();
053      }   
054    
055      /** <!-- begin-user-doc -->
056        * Write your own initialization here
057        * <!-- end-user-doc -->
058      @generated modifiable */
059      private void readObject() {}
060         
061     
062        
063      //*--------------*
064      //* Feature: partOfSpeech
065    
066      /** getter for partOfSpeech - gets The part of speech symbol is stored here.
067       * @generated */
068      public String getPartOfSpeech() {
069        if (CCPTokenAnnotation_Type.featOkTst && ((CCPTokenAnnotation_Type)jcasType).casFeat_partOfSpeech == null)
070          jcasType.jcas.throwFeatMissing("partOfSpeech", "edu.uchsc.ccp.uima.annotation.syntactic.CCPTokenAnnotation");
071        return jcasType.ll_cas.ll_getStringValue(addr, ((CCPTokenAnnotation_Type)jcasType).casFeatCode_partOfSpeech);}
072        
073      /** setter for partOfSpeech - sets The part of speech symbol is stored here. 
074       * @generated */
075      public void setPartOfSpeech(String v) {
076        if (CCPTokenAnnotation_Type.featOkTst && ((CCPTokenAnnotation_Type)jcasType).casFeat_partOfSpeech == null)
077          jcasType.jcas.throwFeatMissing("partOfSpeech", "edu.uchsc.ccp.uima.annotation.syntactic.CCPTokenAnnotation");
078        jcasType.ll_cas.ll_setStringValue(addr, ((CCPTokenAnnotation_Type)jcasType).casFeatCode_partOfSpeech, v);}    
079       
080        
081      //*--------------*
082      //* Feature: stem
083    
084      /** getter for stem - gets The token stem.
085       * @generated */
086      public String getStem() {
087        if (CCPTokenAnnotation_Type.featOkTst && ((CCPTokenAnnotation_Type)jcasType).casFeat_stem == null)
088          jcasType.jcas.throwFeatMissing("stem", "edu.uchsc.ccp.uima.annotation.syntactic.CCPTokenAnnotation");
089        return jcasType.ll_cas.ll_getStringValue(addr, ((CCPTokenAnnotation_Type)jcasType).casFeatCode_stem);}
090        
091      /** setter for stem - sets The token stem. 
092       * @generated */
093      public void setStem(String v) {
094        if (CCPTokenAnnotation_Type.featOkTst && ((CCPTokenAnnotation_Type)jcasType).casFeat_stem == null)
095          jcasType.jcas.throwFeatMissing("stem", "edu.uchsc.ccp.uima.annotation.syntactic.CCPTokenAnnotation");
096        jcasType.ll_cas.ll_setStringValue(addr, ((CCPTokenAnnotation_Type)jcasType).casFeatCode_stem, v);}    
097       
098        
099      //*--------------*
100      //* Feature: lemma
101    
102      /** getter for lemma - gets The token lemma.
103       * @generated */
104      public String getLemma() {
105        if (CCPTokenAnnotation_Type.featOkTst && ((CCPTokenAnnotation_Type)jcasType).casFeat_lemma == null)
106          jcasType.jcas.throwFeatMissing("lemma", "edu.uchsc.ccp.uima.annotation.syntactic.CCPTokenAnnotation");
107        return jcasType.ll_cas.ll_getStringValue(addr, ((CCPTokenAnnotation_Type)jcasType).casFeatCode_lemma);}
108        
109      /** setter for lemma - sets The token lemma. 
110       * @generated */
111      public void setLemma(String v) {
112        if (CCPTokenAnnotation_Type.featOkTst && ((CCPTokenAnnotation_Type)jcasType).casFeat_lemma == null)
113          jcasType.jcas.throwFeatMissing("lemma", "edu.uchsc.ccp.uima.annotation.syntactic.CCPTokenAnnotation");
114        jcasType.ll_cas.ll_setStringValue(addr, ((CCPTokenAnnotation_Type)jcasType).casFeatCode_lemma, v);}    
115       
116        
117      //*--------------*
118      //* Feature: tokenNumber
119    
120      /** getter for tokenNumber - gets The token number indicates the placement of the token within the document text.
121       * @generated */
122      public int getTokenNumber() {
123        if (CCPTokenAnnotation_Type.featOkTst && ((CCPTokenAnnotation_Type)jcasType).casFeat_tokenNumber == null)
124          jcasType.jcas.throwFeatMissing("tokenNumber", "edu.uchsc.ccp.uima.annotation.syntactic.CCPTokenAnnotation");
125        return jcasType.ll_cas.ll_getIntValue(addr, ((CCPTokenAnnotation_Type)jcasType).casFeatCode_tokenNumber);}
126        
127      /** setter for tokenNumber - sets The token number indicates the placement of the token within the document text. 
128       * @generated */
129      public void setTokenNumber(int v) {
130        if (CCPTokenAnnotation_Type.featOkTst && ((CCPTokenAnnotation_Type)jcasType).casFeat_tokenNumber == null)
131          jcasType.jcas.throwFeatMissing("tokenNumber", "edu.uchsc.ccp.uima.annotation.syntactic.CCPTokenAnnotation");
132        jcasType.ll_cas.ll_setIntValue(addr, ((CCPTokenAnnotation_Type)jcasType).casFeatCode_tokenNumber, v);}    
133       
134        
135      //*--------------*
136      //* Feature: typedDependencies
137    
138      /** getter for typedDependencies - gets If this token is known to be involved in any typed dependency structures, then they are linked through this FSArray.
139       * @generated */
140      public FSArray getTypedDependencies() {
141        if (CCPTokenAnnotation_Type.featOkTst && ((CCPTokenAnnotation_Type)jcasType).casFeat_typedDependencies == null)
142          jcasType.jcas.throwFeatMissing("typedDependencies", "edu.uchsc.ccp.uima.annotation.syntactic.CCPTokenAnnotation");
143        return (FSArray)(jcasType.ll_cas.ll_getFSForRef(jcasType.ll_cas.ll_getRefValue(addr, ((CCPTokenAnnotation_Type)jcasType).casFeatCode_typedDependencies)));}
144        
145      /** setter for typedDependencies - sets If this token is known to be involved in any typed dependency structures, then they are linked through this FSArray. 
146       * @generated */
147      public void setTypedDependencies(FSArray v) {
148        if (CCPTokenAnnotation_Type.featOkTst && ((CCPTokenAnnotation_Type)jcasType).casFeat_typedDependencies == null)
149          jcasType.jcas.throwFeatMissing("typedDependencies", "edu.uchsc.ccp.uima.annotation.syntactic.CCPTokenAnnotation");
150        jcasType.ll_cas.ll_setRefValue(addr, ((CCPTokenAnnotation_Type)jcasType).casFeatCode_typedDependencies, jcasType.ll_cas.ll_getFSRef(v));}    
151        
152      /** indexed getter for typedDependencies - gets an indexed value - If this token is known to be involved in any typed dependency structures, then they are linked through this FSArray.
153       * @generated */
154      public CCPTypedDependency getTypedDependencies(int i) {
155        if (CCPTokenAnnotation_Type.featOkTst && ((CCPTokenAnnotation_Type)jcasType).casFeat_typedDependencies == null)
156          jcasType.jcas.throwFeatMissing("typedDependencies", "edu.uchsc.ccp.uima.annotation.syntactic.CCPTokenAnnotation");
157        jcasType.jcas.checkArrayBounds(jcasType.ll_cas.ll_getRefValue(addr, ((CCPTokenAnnotation_Type)jcasType).casFeatCode_typedDependencies), i);
158        return (CCPTypedDependency)(jcasType.ll_cas.ll_getFSForRef(jcasType.ll_cas.ll_getRefArrayValue(jcasType.ll_cas.ll_getRefValue(addr, ((CCPTokenAnnotation_Type)jcasType).casFeatCode_typedDependencies), i)));}
159    
160      /** indexed setter for typedDependencies - sets an indexed value - If this token is known to be involved in any typed dependency structures, then they are linked through this FSArray.
161       * @generated */
162      public void setTypedDependencies(int i, CCPTypedDependency v) { 
163        if (CCPTokenAnnotation_Type.featOkTst && ((CCPTokenAnnotation_Type)jcasType).casFeat_typedDependencies == null)
164          jcasType.jcas.throwFeatMissing("typedDependencies", "edu.uchsc.ccp.uima.annotation.syntactic.CCPTokenAnnotation");
165        jcasType.jcas.checkArrayBounds(jcasType.ll_cas.ll_getRefValue(addr, ((CCPTokenAnnotation_Type)jcasType).casFeatCode_typedDependencies), i);
166        jcasType.ll_cas.ll_setRefArrayValue(jcasType.ll_cas.ll_getRefValue(addr, ((CCPTokenAnnotation_Type)jcasType).casFeatCode_typedDependencies), i, jcasType.ll_cas.ll_getFSRef(v));}
167      }
168    
169
```
